# 145 Assessing guideline concordance of antimicrobials used in Australian long-term care using a national point prevalence audit

**DOI:** 10.1017/ash.2026.10550

**Published:** 2026-06-23

**Authors:** Margaret Fitzpatrick, Charlesnika Evans, Scott Schubert, Marissa Wirth, Laura Scherer

**Affiliations:** 1 VA Eastern Colorado Healthcare System; 2 Northwestern University / Department of Veterans Affairs; 3 Rocky Mountain Regional VA; 4 Center of Innovation for Complex at Edward Hines Jr. VA Hosptial

## Abstract

**Background:** Recurrent urinary tract infections (rUTIs), especially catheter-associated UTIs (CA-UTIs), are common complications in people with neurogenic bladder (NB). The Recurrent UTI Impact Questionnaire (RUTIIQ) includes 5 domains assessing patient-reported quality of life (QoL) impacts from rUTIs: 1.) personal wellbeing, 2.) social wellbeing, 3.) sexual wellbeing, 4.) work and activity interference, and 5.) satisfaction with medical care. The RUTIIQ has only been validated in young women without NB. Our objective was to use cognitive interviews to determine initial adaptations to the RUTIIQ optimizing content validity for people with NB. **Methods:** This study included adult (age < 18) Veterans from two tertiary care Veterans Affairs Medical Centers who had NB due spinal cord injury/disorder (SCI/D) or multiple sclerosis. Virtual cognitive interviews were conducted between June-September 2025 with patients with rUTI, defined as ≥ 2 face-to-face encounters with a UTI diagnosis in the prior 6 months or ≥ 3 in the prior 12 months. Purposeful sampling was used to recruit from both SCI/D and MS groups. Participants were asked to complete the RUTIIQ using “think aloud” technique to evaluate comprehension, understanding and appropriateness. **Results:** Out of 55 eligible patients, 10 interviews were completed. One participant was female (10%), 9 were male (90%). Two participants had MS (20%), 6 had SCI/D (60%). Nine (90%) participants used indwelling or intermittent catheterization. Several participants struggled to focus on UTI-specific impacts, instead responding to items based on QoL and functional impacts from their disability in general. In response, we changed the survey instructions to emphasize that respondents should think about UTI-related impacts and broadened the recall timeframe from 2 weeks to 4 weeks to capture more UTI episodes. The term ‘work’ in the Work and Activity Interference domain was poorly applicable to nearly all participants, whose disability precludes traditional employment. This term was revised to ‘daily activities.’ Finally, several participants noted that the sexual wellbeing domain was not relevant for them, given that sexual dysfunction related to their disability prevented sexual activity. Thus, we added a ‘not applicable’ option to bypass that domain. **Conclusions:** Cognitive interviews with Veterans with NB revealed unique themes primarily related to their underlying disability that affected how rUTIs impact their QoL and functioning. Using these themes, we made substantive changes to the RUTIIQ to adapt it to the NB population. Future work will test the validity and reliability of this adapted RUTIIQ in a national cohort of Veterans with NB.

